# Supplementary material for: Long-term outcomes of baseline grey-zone patients with HBeAg-negative chronic hepatitis B virus infection
Source: JHEP Rep. 2026 Feb 4;8(4):101771. doi: 10.1016/j.jhepr.2026.101771 (PMC13019599; doi:10.1016/j.jhepr.2026.101771)
Supplement: Multimedia component 1 [file mmc1.pdf]

Long-term outcomes of baseline grey-zone patients with HBeAg-negative chronic hepatitis B virus infection

Margarita Papatheodoridi, Sofia Paraskevopoulou, Panagiota Ioannidou, Paraskevi Fytili, Dimitrios S Karagiannakis, Alkistis Papatheodoridi, Stratigoula Sakellariou, Evangelos Cholongitas, Ioannis Vlachogiannakos, George Papatheodoridis

Table of contents

Fig. S1………………………………………………………………………..…2

Fig. S2……………………………………………………………………….4

Fig. S3……………………………………………………………………….5

Table S1……………………………………………………………………...7

Table S2……………………………………………………………………...8

Table S3…………………………………………………………………... 10

Table S4…………………………………………………………..……...11

Table S5……………………………………………………………..........13

Fig. S1. Probability of development of (A) treatment indication or (B) treatment initiation in 1501 patients with HBeAg-negative chronic HBV infection classified in relation to their baseline ALT and/or HBV DNA levels into chronic infection (CIe-: ALT≤ULN and HBVDNA<2000 IU/mL) and grey-zone (GZe-: HBVDNA <2,000 IU/mL and ALT>ULN, HBVDNA 2,000-20,000 IU/mL, or HBVDNA>20,000 IU/mL and ALT<2xULN). All treatments starting during the first year of follow-up were considered to start at year 1. Kaplan-Meier curves were used for estimation of all cumulative rates, which were compared by log-rank test.

Fig. 1A.

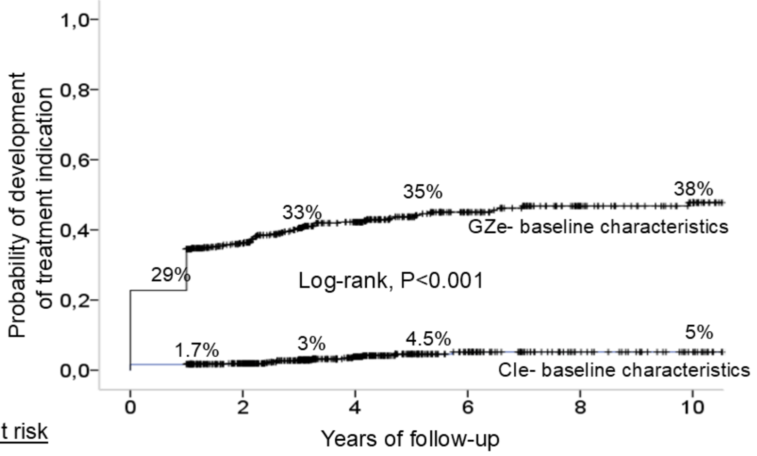

Patients at risk						
CIe-	690	590	315	163	112	90
GZe-	811	469	320	194	131	100

Suppl. Fig. 1A

Fig 1B.

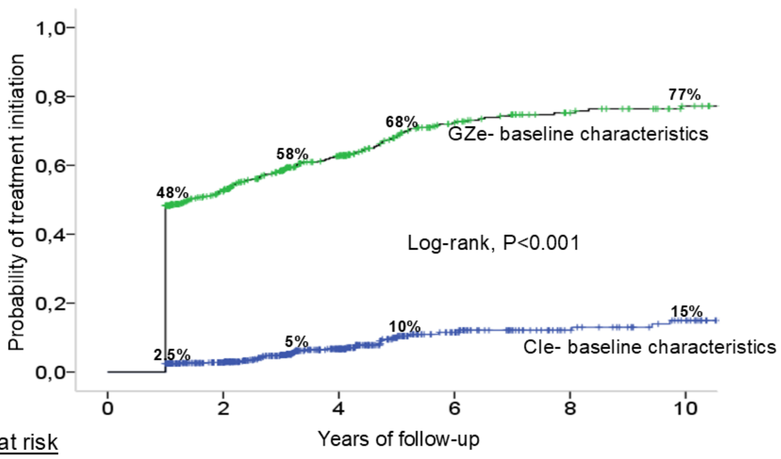

Patients at risk						
Cle-	690	586	304	151	102	82
GZe-	811	322	173	77	44	27

Suppl. Fig. 1B

Fig. S2. Probability of HBsAg loss in 1061 patients with HBeAg-negative chronic HBV infection without treatment indication or initiation during the 1st year in relation to their ALT and/or HBV DNA levels during the same period. Kaplan-Meier curves were used for estimation of all cumulative rates, which were compared by log-rank test.

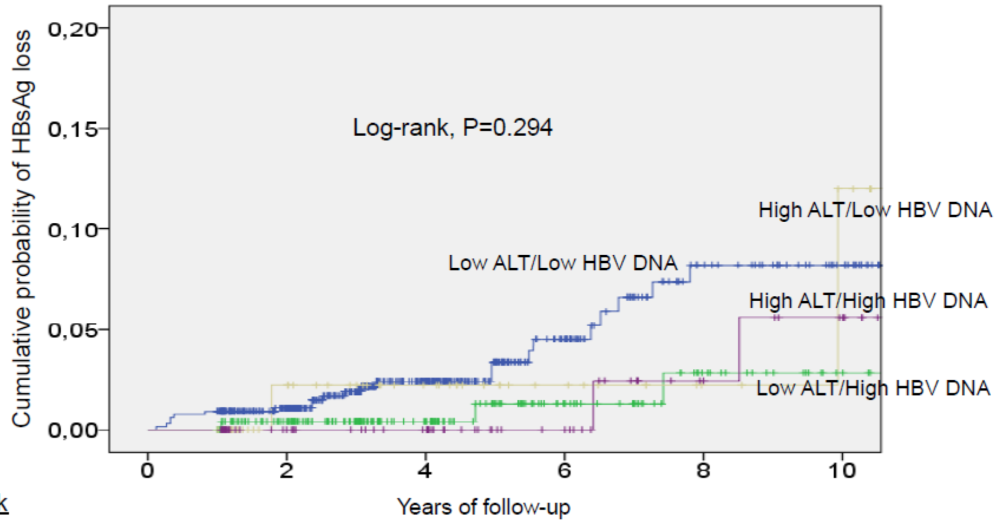

Patients at risk

Low ALT/Low HBV DNA	658	569	301	158	109	88
High ALT/Low HBV DNA	67	44	31	18	11	8
Low ALT/High HBV DNA	246	208	106	85	57	43
High ALT/High HBV DNA	90	74	63	46	32	27

Fig. S3. Probability of (A) hepatocellular carcinoma (HCC) or (B) liver-related event (LRE) development in 1061 patients with HBeAg-negative chronic HBV infection without treatment indication or initiation during the 1st year in relation to their ALT and/or HBV DNA levels during the same period. LRE included HCC, decompensated cirrhosis, liver transplantation and liver-related death. Patients with Low ALT/Low HBV DNA and High ALT/Low HBV DNA as well as those with Low ALT/High HBV DNA and High ALT/High HBV DNA patients had the same numbers and the same cumulative probabilities of HCC and LRE. Kaplan-Meier curves were used for estimation of all cumulative rates, which were compared by log-rank test.

Fig 3A

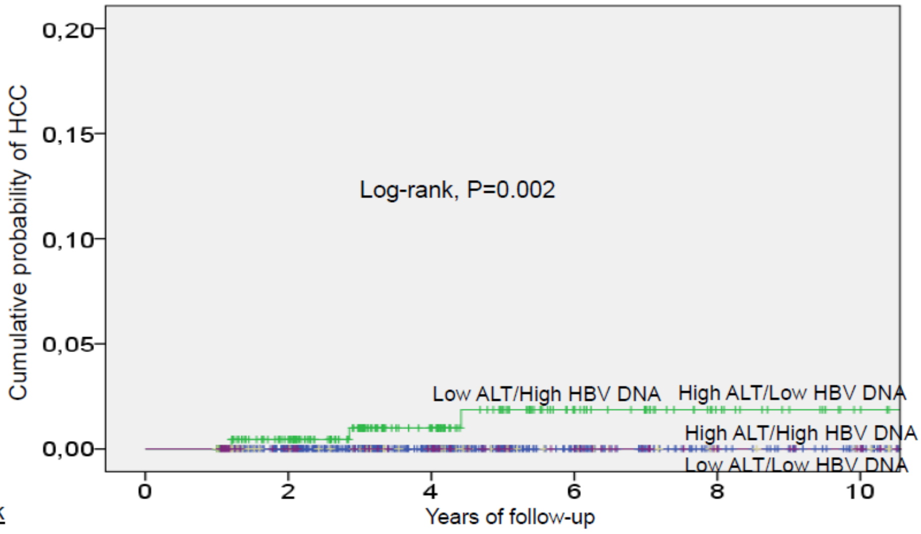

Patients at risk	0	2	4	6	8	10
Low ALT/Low HBV DNA	658	574	309	163	112	91
High ALT/Low HBV DNA	67	45	32	18	11	8
Low ALT/High HBV DNA	246	214	137	86	59	45
High ALT/High HBV DNA	90	75	63	46	33	29

Fig 3B

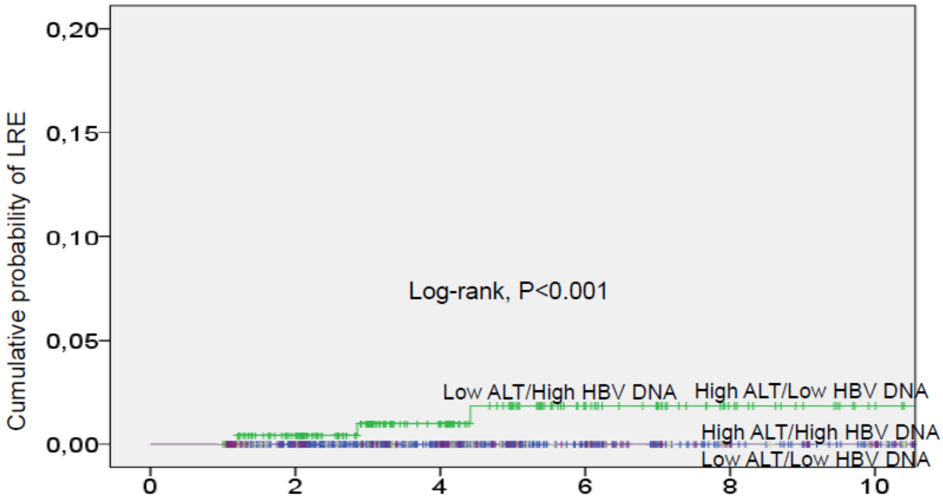

Patients at risk

Low ALT/Low HBV DNA	658	574	308	163	112	91
High ALT/Low HBV DNA	67	45	32	18	11	8
Low ALT/High HBV DNA	246	214	137	86	59	45
High ALT/High HBV DNA	90	73	63	46	33	29

Table S1. Liver fibrosis severity by liver elastography (n=1499) and/or liver biopsy (n=283*) in 1501 HBeAg-negative chronic hepatitis B patients.

Liver stiffness, kPa	**All patients** n (%)	**Patients with liver biopsy*** - Ishak's stage, n (%)				
		0	1	2	3-4	5-6
≤6.4	910 (60.7)	9 (75.0)	45 (68.2)	38 (48.7)	10 (10.3)	0
6.5-7.9	303 (20.2)	3 (25.0)	16 (24.2)	22 (28.2)	27 (27.6)	0
8-9	102 (6.8)	0	4 (6.1)	8 (10.3)	15 (15.5)	0
9.1-12 for ALT>ULN	63 (4.2)	0	1 (1.5)	7 (9.0)	20 (20.6)	2 (7.1)
9.1/12-12.1/15 for ALT≤/>ULN	50 (3.3)	0	0	2 (2.6)	15 (15.5)	0
>12/15 for ALT≤/>ULN	71 (4.7)	0	0	1 (1.3)	10 (10.3)	26 (92.9)
Total	1499 (100)	12 (4.2)	66 (23.5)	78 (27.8)	97 (34.5)	28 (10.0)

*Two patients had a liver biopsy (Ishak's stage: 2 and 6) but not elastography.

ULN: upper limit of normal.

Table S2. Subgroups of 1501 HBeAg-negative chronic hepatitis B (CHBVe-) patients according to their serum HBVDNA and ALT levels at baseline and maximum values during the first year of follow-up.

Maximum HBVDNA (IU/mL) and ALT levels during year-1		Groups of CHBVe- patients by baseline ALT and HBVDNA (IU/mL) levels, n (%)							
		HBVDNA<2,000			HBVDNA-=2,000-20,000			HBVDNA>20,000	
		ALT<ULN	ALT>1-2xULN	ALT>2xULN	ALT<ULN	ALT>1-2xULN	ALT>2xULN	ALT<ULN	ALT>1-2xULN
HBVDNA <2,000	ALT<ULN	677 (45.1)	0	0	0	0	0	0	0
	ALT>1-2xULN	1 (0.1)	58 (3.9)	0	0	0	0	0	0
	ALT>2xULN	0	2 (0.1)	10 (0.7)	0	0	0	0	0
HBVDNA= 2,000-20,000	ALT<ULN	7 (0.5)	0	0	259 (17.3)	0	0	1 (0.1)	0
	ALT>1-2xULN	4 (0.3)	4 (0.3)	0	7 (0.5)	76 (5.1)	0	0	0
	ALT>2xULN	1 (0.1)	0	1 (0.1)	2 (0.1)	10 (0.7)	47 (3.1)	1 (0.1)	1

HBVDNA >20,000	ALT<ULN	0	0	0	1 (0.1)	0	0	54 (3.6)	0
	ALT>1-2xULN	0	0	0	14 (0.9)	10 (0.7)	0	18 (1.2)	131 (8.7)
	ALT>2xULN	0	0	0	8 (0.5)	9 (0.6)	18 (1.2)	14 (0.9)	56 (3.7)
Total		690 (46.0)	64 (4.3)	11 (0.7)	291 (19.4)	105 (7.0)	65 (4.3)	88 (5.9)	187 (12.5)

ULN: upper limit of normal

Table S3. Development of treatment indications and treatment initiation during the first year of follow-up in 1501 HBeAg-negative chronic hepatitis B patients according to their maximum serum HBVDNA and ALT levels during the same period. Treatment indication was considered to be met in patients with a) ALT>2xULN and HBVDNA>20,000 IU/mL regardless of histological severity, b) HBVDNA>2,000 IU/mL and advanced fibrosis regardless of ALT and c) detectable HBVDNA and cirrhosis.

Maximum HBVDNA (IU/mL) and ALT levels during year-1		Treatment indication, n/N (%)	Treatment initiation, n (%)
HBVDNA <2,000	ALT≤ULN	10/677 (1.5)	14/677 (2.1)
	ALT>1-2xULN	1/59 (1.7)	2/59 (3.4)
	ALT>2xULN	1/12 (8.3)	2/12 (16.7)
HBVDNA= 2,000-20,000	ALT≤ULN	21/267 (7.9)	37/267 (13.9)
	ALT>1-2xULN	20/91 (22.0)	28/91 (30.8)
	ALT>2xULN	26/62 (41.9)	49/62 (79.0)
HBVDNA >20,000	ALT≤ULN	11/55 (20.0)	25/55 (45.5)
	ALT>1-2xULN	50/173 (28.9)	147/173 (75.0)
	ALT>2xULN	105/105 (100.0)	105/105 (100.0)
Total		245/1501 (16.3)	409/1501 (27.2)

ULN: upper limit of normal

Table S4. Multivariable Cox regression analyses for factors associated with the main outcomes in in 1061 patients with HBeAg-negative chronic HBV infection (CHBVe-) without treatment indications and initiation during year-1. Main outcomes include HBsAg loss, hepatocellular carcinoma and any liver related event (LRE).

	**HBsAg loss**	**Hepatocellular carcinoma or LRE**
	Adjusted HR (95% CI), P	Adjusted HR (95% CI), P
Age, per year	NA	1.09 (1.01-1.18), 0.032
Gender, male vs female	NA	NA
Origin, Greece vs abroad	NA	NS
Body mass index, per kg/m^2	1.15 (1.06-1.25), 0.001	NA
Diabetes, yes vs no	NA	NA
ALT, per IU/L	NA	NA
AST, per IU/L	NA	NA
Platelets, per 10^3/mm^3	NA	NA
HBVDNA, per log$_{10}$ IU/mL	0.71 (0.58-0.88), 0.002	NS

CHBVe- group, Grey-Zone vs Chronic infection	NA	NA
Treatment as time dependent variable	NA	NS

HR: Hazard ratio, CI: confidence interval, ALT: alanine aminotransferase, AST: aspartate aminotransferase, NA: not applicable (P>0.10 in univariable Cox regression analysis); NS: non-significant in multivariable Cox regression analysis (P>0.10).

Table S5. Multivariable Cox regression analyses for baseline factors associated with the main outcomes in 1501 patients with HBeAg-negative chronic HBV infection (CHBVe-). Main outcomes include HBsAg loss, hepatocellular carcinoma and any liver related event (LRE). Patients were censored at the onset of treatment.

	**HBsAg loss**	**Hepatocellular carcinoma**	**Any LRE**
	Adjusted HR (95% CI), P	Adjusted HR (95% CI), P	Adjusted HR (95% CI), P
Age, per year	NA	1.08 (1.02-1.15), 0.015	1.09 (1.02-1.17), 0.011
Gender, male vs female	NA	NA	NA
Origin, Greece vs abroad	NA	NA	NA
Body mass index, per kg/m^2	1.17 (1.07-1.27), 0.001	NA	NA
Diabetes, yes vs no	NA	NA	NS
ALT, per IU/L	NA	NA	NS
AST, per IU/L	NA	NA	NS
Platelets, per $10^3/mm^3$	NA	NS	NS
HBVDNA, per log_{10} IU/mL	0.64 (0.48-0.75), 0.002	2.06 (1.20-3.52), 0.009	1.65 (1.03-2.64), 0.036

CHBVe- group, Grey-Zone vs Chronic infection	NS	NA	NA

HR: Hazard ratio, CI: confidence interval, ALT: alanine aminotransferase, AST: aspartate aminotransferase, NA: not applicable (P>0.10 in univariable Cox regression analysis); NS: non-significant in multivariable Cox regression analysis (P>0.10).
