# Supplementary material for: Long-term outcomes of baseline grey-zone patients with HBeAg-negative chronic hepatitis B virus infection
Source: JHEP Rep. 2026 Feb 4;8(4):101771. doi: 10.1016/j.jhepr.2026.101771 (PMC13019599; doi:10.1016/j.jhepr.2026.101771)
Supplement: Multimedia component 4 [file mmc4.pdf]

Research article

JHEP Reports

Long-term outcomes of baseline grey-zone patients with HBeAg-negative chronic hepatitis B virus infection

Authors

Margarita Papatheodoridi, Sofia Paraskevopoulou, Panagiota Ioannidou, …, Evangelos Cholongitas, Ioannis Vlachogiannakos, George Papatheodoridis

Correspondence

gepapath@med.uoa.gr (G. Papatheodoridis).

Graphical abstract

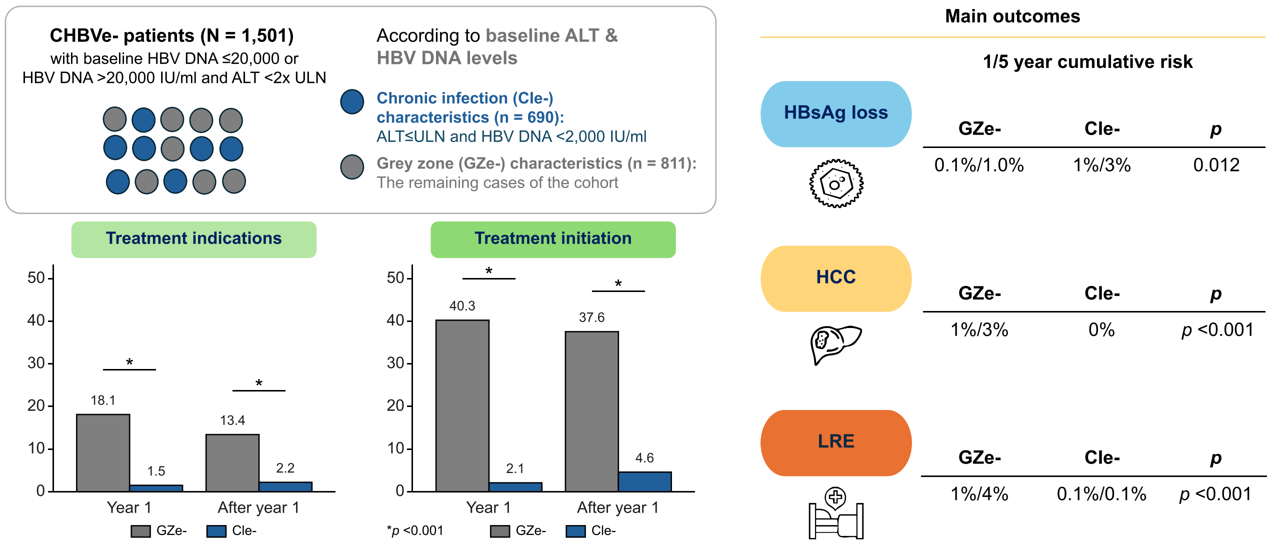

Highlights:

- Outcomes of grey-zone HBeAg-negative chronic HBV remain uncertain, hindering optimal management and timely treatment.
- Real-world data from an expert HBV center provide insights to guide management of grey-zone HBeAg-negative patients.
- Grey-zone HBeAg-negative patients are a large HBV group at higher risk of cancer and liver-related events.

Impact and implications:

The outcomes of patients with grey-zone HBeAg-negative chronic HBV infection remain uncertain, hindering their optimal management and timely treatment in clinical practice. Our real-world data from a tertiary HBV center provide valuable insights to guide the management of this patient group. Grey-zone HBeAg-negative patients represent a substantial proportion of the chronic HBV population and require careful monitoring, as they are at increased risk of hepatocellular carcinoma and other liver-related events.

https://doi.org/10.1016/j.jhepr.2026.101771

© 2026 The Author(s). Published by Elsevier B.V. on behalf of European Association for the Study of the Liver (EASL). This is an open access article under the CC BY license (http://creativecommons.org/licenses/by/4.0/). JHEP Reports, 2026, 8, 1–11

JHEP|Reports

Long-term outcomes of baseline grey-zone patients with HBeAg-negative chronic hepatitis B virus infection

Margarita Papatheodoridi[1], Sofia Paraskevopoulou[1], Panagiota Ioannidou[1], Paraskevi Fytili[1], Dimitrios S. Karagiannakis[1], Alkistis Papatheodoridi[1], Stratigoula Sakellariou[2], Evangelos Cholongitas[1], Ioannis Vlachogiannakos[1], George Papatheodoridis[1,*]

JHEP Reports **2026**. vol. 8 | 1–11

Check for updates

Background & Aims: The optimal management and outcomes of patients with HBeAg-negative grey-zone (GZe-) chronic HBV infection remain debatable. We assessed the outcomes and real-life management of GZe-patients and compared them to patients with typical HBeAg-negative chronic infection (CIe-).

Methods: We included all HBeAg-negative patients with baseline HBV DNA ≤20,000 IU/ml or HBV DNA >20,000 IU/ml and ALT <2x the upper limit of normal (ULN). Among patients without treatment indications in year 1, those with persistently normal ALT (≤ULN) and HBV DNA <2,000 IU/ml were defined as typical CIe-, and all others were classified as GZe-. Outcomes included treatment initiation, HBsAg loss, hepatocellular carcinoma (HCC), and liver-related events (LREs: HCC, decompensated cirrhosis, liver transplantation, or liver-related death).

Results: In total, 1,501 patients with a mean follow-up of 6.0 ± 4.6 years were included (GZe-/CIe-baseline characteristics: 811/690; GZe-/CIe-at year 1: 719/677). Compared with CIe-patients, GZe-patients more frequently developed treatment indications after year 1 (year 5: 13.4% *vs.* 2.2%; log-rank, *p* <0.001) and were more frequently treated after year 1 (year 5: 37.6% *vs.* 4.6%; log-rank, *p* <0.001). GZe-patients, particularly those with GZe-baseline characteristics, were less likely to achieve HBsAg loss (1-/5-year: 0.1/1.0% *vs.* 1/3%; log-rank, *p* = 0.012) and more frequently developed HCC (1-/5-year: 1/3% *vs.* 0%; log-rank, *p* <0.001) and LREs (1-/5-year: 1/4% *vs.* 0.1%; log-rank, *p* <0.001).

Conclusions: GZe-patients represent a large proportion of patients with chronic HBV seen at tertiary centers and meet treatment indications more frequently than CIe-patients. They also have a lower probability of HBsAg loss and higher risks of HCC and LREs despite treatment initiation in most cases; therefore, their optimal management and timing of treatment initiation require further evaluation.

© 2026 The Author(s). Published by Elsevier B.V. on behalf of European Association for the Study of the Liver (EASL). This is an open access article under the CC BY license (http://creativecommons.org/licenses/by/4.0/).

Introduction

While the natural history of chronic infection with hepatitis B virus (HBV) has been well defined,[1] indications for treatment remain complicated mainly because HBV cannot be eradicated.[2–5] Current treatment indications aim to identify patients with chronic HBV infection who have disease activity and/or liver fibrosis, and are often based not only on alanine aminotransferase (ALT) and HBV DNA levels but also on the severity of liver histological lesions.[2–5] Although recent guidelines recommend initiation of therapy in patients with HBV DNA and ALT above certain levels regardless of liver histology,[3–5] a variable proportion of patients with chronic HBV infection remain as indeterminate or grey-zone (GZ) cases after assessment of ALT and HBV DNA levels.[6–9]

Patients with GZ chronic HBV infection can be further classified into HBeAg-positive and HBeAg-negative (GZe-) groups, which differ in epidemiological and virological characteristics – typically high *vs.* low HBV DNA levels, respectively – and present distinct challenges regarding optimal management.[6,8] Given that most patients with chronic HBV infection are HBeAg-negative,[1] GZe-represent the predominant GZ subgroup.[6–8] For GZe-cases, it is still unclear whether therapy is warranted or if a more benign disease course is expected, so their optimal management remains controversial.[10] Along these lines, there are different recommendations in different scientific guidelines. Namely, the European Association for the Study of the Liver (EASL) guidelines do not recommend treatment initiation in all GZe-cases but only in those fulfilling certain criteria of histological severity.[3] In contrast, treatment is recommended for all GZe-cases in the latest guidelines from both China and the World Health Organization in an effort to treat all and simplify treatment indications for clinicians and patients, thus preventing future negative outcomes by starting therapy earlier.[11,12] Given that GZe-cases represent a large proportion of patients with chronic HBV infection seen in our clinical practice, we aimed to evaluate the outcomes and real-life management of GZe-patients and compare them to patients with typical HBeAg-negative chronic infection (CIe-), also known as "inactive carriers".

* Corresponding author. Address: 1st Department of Gastroenterology, Medical School of National and Kapodistrian University of Athens, General Hospital of Athens "Laiko", 17 Agiou Thoma street, 11527 Athens, Greece. Tel: +302132061115.
E-mail address: gepapath@med.uoa.gr (G. Papatheodoridis).
https://doi.org/10.1016/j.jhepr.2026.101771

Patients and methods

Patient population

This retrospective cohort study included all adult (≥18 years) patients with HBeAg-negative chronic infection who had at least one visit to our liver center between 2010 and 2019 and did not meet the EASL treatment criteria based on initial HBV DNA and ALT levels (HBV DNA >20,000 IU/ml and ALT >2x the upper limit of normal [ULN]).[3] In particular, all included patients had either (a) HBV DNA ≤20,000 IU/ml, or (b) HBV DNA >20,000 IU/ml with ALT ≤2x ULN at baseline. Patients with coinfection(s), use of immunosuppression, liver decompensation, hepatocellular carcinoma (HCC), alcohol abuse (mean daily alcohol consumption >60/40 g for males/females) or other causes of liver injury were excluded. The study protocol was approved by our hospital ethics committee. Given the anonymous use of retrospective data and patients' general consent for research purposes, the requirement for specific informed consent was waived.

Definitions

Patients were initially classified into two groups according to their baseline ALT and HBV DNA levels: a) patients with CIe-baseline characteristics: low ALT (≤ULN) and low HBV DNA (<2,000 IU/ml) and b) patients with GZe-baseline characteristics: high ALT (>ULN) and/or high HBV DNA (≥2,000 IU/ml). Patients were further classified into four subgroups: low-ALT/low-DNA, high-ALT/low-DNA, low-ALT/high-DNA and high-ALT/high-DNA.

Advanced liver fibrosis was diagnosed in patients with liver stiffness of 9.1-12 or 12.1-15 kPa in case of ALT ≤ULN or >ULN, respectively,[13] and/or histological Ishak's stage 3-4 fibrosis. Cirrhosis was diagnosed in patients with liver stiffness of >12 or >15 kPa in case of ALT ≤ULN or >ULN, respectively,[13] and/or histological Ishak's stage 5-6 fibrosis. HCC was diagnosed by histological and/or standard radiological criteria.[14] Liver-related events (LREs) were defined as HCC, decompensated cirrhosis, liver transplantation or liver-related death.

Based on the 2017 EASL guidelines,[3] treatment indications were considered to be met in patients with a) ALT >2xULN and HBV DNA >20,000 IU/ml regardless of histological severity, b) HBV DNA >2,000 IU/ml and advanced fibrosis regardless of ALT and c) detectable HBV DNA and cirrhosis.

Patients without treatment indications during the first year were considered as typical CIe-cases if they maintained ALT ≤ULN and HBV DNA <2,000 IU/ml at all visits and as GZe-cases if they developed ALT >ULN and/or HBV DNA ≥2,000 IU/ml at any visit.

Follow-up

Patients were followed every 3–4 months during year 1 and every 6–12 months thereafter, according to standard clinical practice. For this study, patient data were recorded every 12 months unless higher ALT and/or HBV DNA levels were observed in the interim. Each visit included clinical examination and determination of at least ALT, aspartate aminotransferase (AST) and HBV DNA levels. The ULN for ALT/AST was 40 IU/L. All patients with cirrhosis and those with PAGE-B ≥10[15] were recommended to undergo HCC surveillance with ultrasonography (usually combined with alpha-fetoprotein) every 6 months.

The severity of histological lesions was assessed by liver elastography and/or biopsy, which were performed within the first 6 months. Liver elastography was repeated every ≤2 years. Antiviral treatment was initiated at the discretion of the treating physicians.

Study endpoints

Primary endpoints were the main outcomes including HBsAg loss, HCC and LRE. Treatment initiation within or beyond year 1 was considered as a secondary endpoint.

Statistical analysis

All data were entered into a specifically designed SPSS database. Statistical analyses were performed using SPSS package (IBM® SPSS® Statistics 29.0, SPSS Inc, IBM, Chicago, IL, USA). Quantitative variables were expressed as means ± SD or medians (IQR) and were compared by t-test or Mann-Whitney test. Corrected chi-square or two-sided Fisher's exact tests were used to test for associations between two categorical variables. Kaplan-Meier curves were used for estimation of cumulative rates, which were compared by log-rank test. Treatment starting at any time during year 1 was considered to start at year 1. Cox proportional hazards regression models were used to estimate the effect of variables on the hazard of the main outcomes. Cox regression time-dependent analyses were used to assess the effect of treatment initiation on the main study outcomes. Multivariable Cox proportional hazards models including all factors with significant or trends (p <0.10) for associations in univariable analyses were used to identify independent prognostic factors. Hazard ratios (HR) and their 95% CIs along with p values are presented. Similar Cox regression analyses were also performed with treated patients censored at the onset of treatment. p <0.05 was considered statistically significant.

Results

Main patient characteristics

Of 2,012 patients with chronic HBV followed between 2010-2019, 1,501 (74.6%) were included. Of the 511 excluded cases, 82 were HBeAg-positive, 398 were HBeAg-negative with baseline ALT and HBV DNA levels meeting criteria for immediate treatment initiation, and 31 met other exclusion criteria.

Of the 1,501 included patients, 690 (46%) had CIe- and 811 (54%) had GZe-baseline characteristics (Table 1). The mean follow-up was 6.0 ± 4.6 years. Patients with CIe-baseline characteristics did not differ from those with GZe-in age, BMI, or prevalence of diabetes; however, they were less frequently male (56% *vs.* 64%, p = 0.002) and, as expected, had lower ALT, AST, and HBV DNA levels and higher platelet counts (all p values <0.001). Of the 811 patients with GZe-baseline characteristics, 291 (35.9%) had ALT ≤ULN and HBV DNA 2,000–20,000 IU/ml; 88 (10.9%) had ALT ≤ULN and HBV DNA >20,000 IU/ml; 64 (7.9%) had ALT >1–2x ULN and HBV DNA <2,000 IU/ml; 105 (12.9%) had ALT >1–2x ULN and HBV DNA 2,000–20,000 IU/ml; 187 (23.1%) had ALT >1–2x ULN and HBV DNA >20,000 IU/ml; 11 (1.4%) had ALT >2x ULN and HBV DNA <2,000 IU/ml; and 65 (8.0%) had ALT >2x ULN and HBV DNA 2,000–20,000 IU/ml.

Table 1. Main baseline characteristics of 1,501 HBeAg-negative patients according to their baseline ALT and HBV DNA characteristics (chronic infection and grey-zone).

	All patients (N = 1,501)	Chronic infection (n = 690)	Grey-zone (n = 811)	*p* value
Age, years	45 ± 14	45 ± 14	45 ± 14	0.788[1]
Male sex, n (%)	908 (60.5)	387 (56)	521 (64)	0.001[2]
Country of origin, n (%)				0.474[2]
Greece	860 (57)	388 (56)	472 (58)	
Abroad	641 (43)	302 (44)	339 (42)	
Body mass index, kg/m^2	25.8 ± 3.5	25.7 ± 3.4	25.8 ± 3.5	0.628[1]
Diabetes, n (%)	72 (4.8)	28 (4.1)	44 (5.4)	0.265[2]
ALT, n (%)				<0.001[2]
≤ULN	1,066 (71)	690 (100)	376 (46)	
1-2x ULN	356 (24)		356 (44)	
>2x ULN	79 (5)		79 (10)	
ALT, IU/L	28 (26)	22 (12)	44 (42)	<0.001[3]
AST, IU/L	29 (14)	21 (7)	30 (20)	<0.001[3]
Platelets, x10^3/mm^3	219 ± 47	227 ± 44	212 ± 48	<0.001[1]
HBV DNA, n (%)				<0.001[2]
<2,000 IU/ml	765 (51)	690 (100)	75 (9)	
2,000-20,000 IU/ml	461 (31)		461 (57)	
>20,000 IU/ml	275 (18)		275 (34)	
Follow-up, years	6.0 ± 4.6	5.1 ± 4.1	6.8 ± 4.8	<0.001[1]

Quantitative variables are expressed as mean ± SD or median (IQR) and were compared between groups by [1]t-test or Mann-Whitney test,[3] respectively. Qualitative variables were compared between groups by corrected chi-squared test.[2] ALT, alanine aminotransferase; AST, aspartate aminotransferase; ULN, upper limit of normal.

First year of follow-up

During year 1, 245 (16.3%) patients developed treatment indications, 593 (39.5%) were characterized as GZe- and 663 (44.2%) as CIe-patients. Liver fibrosis severity was assessed in all patients by month 6 using liver elastography (n = 1,499) and/or biopsy (n = 283) (Table S1). After fibrosis severity assessment, treatment indications (HBV DNA >2,000 and advanced fibrosis or detectable HBV DNA and cirrhosis) were met by 176/1,501 (11.7%) patients and were more frequent in patients with GZe-than CIe-baseline characteristics (165/811 [20.3%] *vs.* 11/690 [1.6%], *p* <0.001). In particular, treatment indications based on fibrosis severity were observed more frequently in patients with baseline ALT >2x ULN (25/79 [31.6%]) than >1-2x ULN (78/356 [21.9%]) or ≤ULN (73/1,066 [6.8%]) (*p* <0.001) and with HBV DNA >20,000 (75/275 [27.3%]) than 2,000-20,000 (87/461 [18.9%]) or <2,000 IU/ml (14/765 [1.8%] (*p* <0.001). The highest treatment indication rate was observed in patients with ALT >1-2x ULN and HBV DNA >20,000 (24/65 [36.9%]). In multivariable logistic regression analysis, baseline ALT and HBV DNA levels were independently associated with treatment indications (data not shown).

During year 1, there were changes in HBV DNA and ALT levels in some patients (Table S2), leading to development of treatment indications in another 69/1,501 (4.6%) patients (all with GZe-baseline characteristics) (Table S3). Regarding baseline values, treatment indications during year 1 developed in 11/690 (1.8%) patients with baseline low-ALT/low-DNA, 3/75 (4.0%) with high-ALT/low-DNA, 70/379 (18.5%) with low-ALT/high-DNA, and 161/357 (45.1%) with high-ALT/high-DNA (*p* <0.001).

Treatment was initiated in 409/1,501 (27.2%) patients within year 1: 214/245 (87.3%) patients who developed treatment indications, 186/593 (31.4%) GZe- and 9/663 (1.4%) CIe-patients (*p* <0.001). Treatment was initiated during year 1 in 17/690 (2.5%) patients with baseline low-ALT/low-DNA, 7/75 (9.3%) with high-ALT/low-DNA, 121/379 (31.9%) with low-ALT/high-DNA and 264/357 (73.9%) with high-ALT/high-DNA levels (*p* <0.001). The treatment initiation rates in relation to maximum ALT and HBV DNA levels during year 1 are presented in Table S3. In multivariable logistic regression analysis, higher baseline ALT and HBV DNA levels, as well as baseline high-ALT/high-DNA, were independently associated with treatment indications or initiation during year 1 (data not shown).

Treatment indications after year 1

Among the 1,092 patients without treatment during year 1, treatment indications developed in 10/654 (1.5%) CIe- and 47/407 (11.5%) GZe-cases (p <0.001), while another 31 patients had treatment indications but did not initiate treatment during year 1. Moreover, during follow-up, 62/654 (9.5%) CIe-patients at year 1 developed ALT and/or HBV DNA values consistent with the GZe-group, while 133/288 (46.2%) untreated GZe-patients at year 1 transitioned to CIe-characteristics.

The cumulative rates of treatment indication after year 1 were higher in GZe-than CIe-patients (3-, 5- and 10-year: 8.2%, 13.4% and 19.4% *vs.* 1.2%, 2.2% and 2.2%, *p* <0.001). These rates were highest in patients with high ALT/high HBV DNA at year 1, intermediate in those with low ALT/high HBV DNA or high ALT/low HBV DNA, and lowest in those with low ALT/low HBV DNA (5-year: 25.5% *vs.* 10.4% or 7.8% *vs.* 2.3%, respectively; *p* <0.001) (Fig. 1A). In multivariable analyses, factors independently associated with treatment indication after year 1 included male sex, higher baseline ALT and HBV DNA levels, lower platelets and GZe-group (Table 2).

Treatment initiation after year 1

Treatment was initiated in 170 (11.3%) patients beyond year 1. Treatment beyond year 1 was initiated more frequently in GZe-than CIe-patients (121/407 [29.7%] *vs.* 24/654 [3.7%], *p* <0.001), as well as in 25/31 (80.6%) patients with treatment indications during year 1.

Treatment was initiated in 297/306 (97.1%) patients who developed treatment indications at any time, 257/604 (42.5%) patients with GZe-characteristics at any time and 25/591 (4.2%) patients always maintaining CIe-characteristics (*p*

A

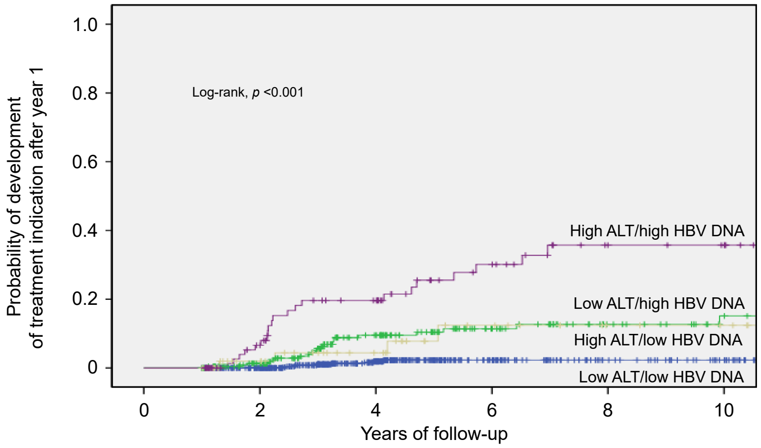

N° at risk						
Low ALT/low HBV DNA	658	574	304	158	108	87
High ALT/low HBV DNA	67	44	31	17	10	8
Low ALT/high HBV DNA	246	212	125	75	48	35
High ALT/high HBV DNA	90	69	50	30	17	14

B

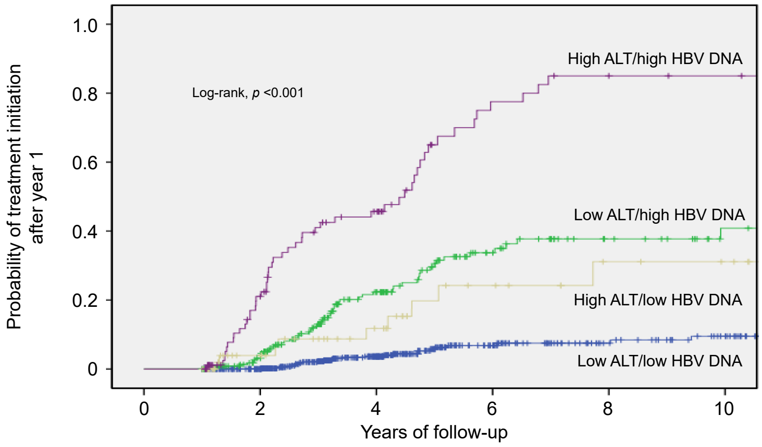

N° at risk						
Low ALT/low HBV DNA	658	574	296	148	99	80
High ALT/low HBV DNA	67	42	28	15	9	7
Low ALT/high HBV DNA	246	208	106	53	30	19
High ALT/high HBV DNA	90	59	33	9	4	3

Fig. 1. Probability of treatment indication or treatment initiation after year-1 in 1,061 HBeAg-negative patients without treatment indication or initiation during year 1 in relation to ALT and HBV DNA levels during the same period. Kaplan-Meier curves were used for estimation of all cumulative rates, which were compared by log-rank test. ALT, alanine aminotransferase.

Table 2. Multivariable Cox regression analyses for the development of treatment indications and for treatment initiation after year 1 in 1,061 HBeAg-negative patients without treatment indications and initiation during year 1.

	Treatment indications Adjusted HR (95% CI), *p* values	Treatment initiation Adjusted HR (95% CI), *p* values
Age, per year	NA	NA
Sex, male *vs.* female	2.01 (1.09-3.74), 0.027	1.71 (1.18-2.46), 0.004
Origin, Greece *vs.* abroad	NA	NA
Body mass index, per kg/m^2	NA	NA
Diabetes, yes *vs.* no	NA	NA
ALT, per IU/L	1.019 (1.007-1.031), 0.001	1.015 (1.008-1.022), <0.001
AST, per IU/L	n.s.	n.s.
Platelets, per 10^3/mm^3	n.s.	0.995 (0.991-0.999), 0.016
HBV DNA, per log$_{10}$ IU/ml	1.43 (1.07-1.91), 0.017	1.78 (1.45-2.23), <0.001
Grey-zone *vs.* chronic infection	2.95 (1.27-6.85), 0.012	2.98 (1.75-5.08), <0.001

ALT, alanine aminotransferase; AST, aspartate aminotransferase; HR, hazard ratio; NA, not applicable (*p* >0.10 in univariable Cox regression analysis); n.s., non-significant in multivariable Cox regression analysis (*p* >0.10).

<0.001). Initial treatment was nucleos(t)ide analogue (NA) monotherapy in 552/579 (95.3%), pegylated interferon alfa-2a (peg-IFNa) monotherapy in 11/579 (1.9%) and combination of NAs plus peg-IFNa in another 16/579 (2.8%) patients.

The 3-, 5- and 10-year cumulative rates of treatment initiation were higher in GZe- (18.5%, 37.6% and 52.6%) compared to CIe- (2.2%, 4.6% and 7.3%; log-rank, p <0.001) patients. Specifically, these rates were highest in patients with high-ALT/high-DNA at year 1, intermediate in those with low-ALT/high-DNA and high-ALT/low-DNA, and lowest in those with low-ALT/low-DNA (5-year: 65.0% *vs.* 29.6% or 19.8% *vs.* 5.8%, respectively; p <0.001) (Fig. 1B). In multivariable analyses, factors independently associated with treatment initiation after year 1 included male sex, higher baseline ALT and HBV DNA levels and GZe-group (Table 2).

The probabilities of treatment indication or initiation are shown in Fig. S1.

Main outcomes

HBsAg loss was observed in 19/811 (2.3%) patients with GZe- and 24/690 (3.5%) patients with CIe-baseline characteristics or 11/407 (2.7%) GZe- and 23/654 (3.5%) CIe-patients without treatment initiation at year 1. The 3-, 5- and 10-year cumulative HBsAg loss rates were higher in patients with CIe- (1.8%, 3% and 8%) compared to GZe-baseline characteristics (0.4%, 1.0% and 4.2%; log-rank, p = 0.012) (Fig. 2A), as well as in patients with baseline low-ALT/low-DNA compared to low-ALT/high-DNA (p = 0.034) or high-ALT/high-DNA (p = 0.021) but not compared to high-ALT/low-DNA (p = 0.771); HBsAg loss rates did not differ among patient subgroups with high-ALT and/or high-DNA (Fig. 2B). Cumulative HBsAg loss rates were relatively lower in GZe-than CIe-patients (3-, 5-, 10-year rates: 0.8%, 1.1%, 4.4% *vs.* 1.9%, 3.4%, 8.3%; log-rank, p = 0.073), while they did not differ among the year-1 ALT/HBV DNA subgroups (data not shown). In univariable analyses, HBsAg loss was associated with greater BMI, CIe-baseline characteristics and no treatment as a time-dependent variable. In multivariable analysis, HBsAg loss was associated only with greater BMI and had a trend for association with CIe-baseline characteristics (Table 3).

HCC was diagnosed only in patients with GZe-baseline characteristics (32/811 [4%]) with 5-/10-year cumulative rates of 3%/5% (log-rank, p <0.001 *vs.* CIe-baseline characteristics) (Fig. 3A) or only in GZe-patients (15/593 [2.5%]; log-rank, p <0.001 *vs.* CIe-). Cumulative HCC rates were lower in patients with baseline low-ALT/low-DNA compared to high-ALT/low-DNA (p = 0.002) or low-ALT/high-DNA (p <0.001) or high-ALT/high-DNA (p <0.001), but they did not differ among patient subgroups with high-ALT and/or high-DNA (Fig. 3B). HCC development was associated with age, sex, origin, diabetes, baseline AST, platelets and HBV DNA levels, as well as with GZe-baseline characteristics or group and treatment as a time-dependent variable (data not shown). In multivariable analysis, the probability of HCC was independently associated with older age, male sex, lower baseline platelets and GZe-baseline characteristics or group (Table 3). HCC development was not significantly associated with HBsAg loss, although none of the 43 patients who achieved HBsAg loss developed HCC.

Any LRE occurred in 36/811 (4.4%) patients with GZe-baseline characteristics (5-/10-year cumulative rates: 4%/6%) and only 1/690 (0.1%) patients with CIe-baseline characteristics (log-rank, p <0.001) (Fig. 4A) or in 18/593 (3.0%) GZe- and none of the 663 CIe-patients (log-rank, p <0.001). Cumulative LRE rates were lower in patients with baseline low-ALT/low-DNA compared to high-ALT and/or high-DNA (all p <0.001), but they did not differ among patient subgroups with high-ALT and/or high-DNA (Fig. 4B). LRE development was associated with age, sex, origin, presence of diabetes, baseline ALT, AST, platelets and HBV DNA levels, GZe-baseline characteristics or group and treatment as a time-dependent variable (data not shown). In multivariable analysis, the probability of LREs was independently associated with older age, lower platelets, GZe-baseline characteristics or group and treatment as a time-dependent variable (Table 3).

Main outcomes in specific patient subgroups

HCC developed more frequently in patients aged >50 (29/560) than ≤50 years (3/941) (3-, 5-, 10-year cumulative rate: 2.1%, 4.1%, 7.1% *vs.* 0%, 0.4%, 0.4%, respectively; p <0.001). Similarly, LREs developed more frequently in patients aged >50 (34/560) than ≤50 years (3/941) (3-, 5-, 10-year cumulative rate: 2.5%, 5.0%, 8.0% *vs.* 0%, 0.4%, 0.4%, respectively; p <0.001). In contrast, there was no difference in HBsAg loss rates between patients aged >50 (19/560) and ≤50 years (24/941) (log-rank, p = 0.653).

HBsAg loss was observed more frequently in patients with baseline HBV DNA <2,000 IU/ml (27/765) *vs.* 2,000-20,000 IU/ml (7/461) or >20,000 IU/ml (9/275) (3-, 5-, 10-year cumulative rates: 1.8%, 3.1%, 8.0% *vs.* 0.7%, 1.1%, 1.7% or 0.4%, 0.4%, 6.7%, respectively; p = 0.009). HCC developed less frequently in patients with baseline HBV DNA <2,000 IU/ml (1/765) *vs.* 2,000-20,000 IU/ml (13/461) or >20,000 IU/ml (18/275) (3-, 5-, 10-year cumulative rate: 0.1%, 0.1%, 0.1% *vs.* 1.0%, 3.2%, 4.9% *vs.* 2.3%, 3.4%, 5.7%, respectively; p <0.001). Similarly, LREs developed less frequently in patients with baseline HBV DNA <2,000 IU/ml (4/765) *vs.* 2,000-20,000 IU/ml (15/461) or >20,000 IU/ml (18/275) (3-, 5-, 10-year cumulative rate: 0.1%, 0.6%, 0.6% *vs.* 1.4%, 3.6%, 5.3% *vs.* 2.3%, 3.4%, 5.7%, respectively; p <0.001). In patients with baseline HBV DNA ≤20,000 IU/ml, 3-, 5-, 10-year cumulative rates were 1.4%, 2.2%, 4.9% for HBsAg loss, 0.5%, 1.5%, 2.4% for HCC and 0.6%, 2%, 2.9% for LREs, respectively.

In 1,061 patients without treatment indications and/or initiation during year 1, HBsAg loss was observed in 34 (3.2%) (3-, 5-, 10-year cumulative rates: 1.4%, 3.4%, 6.7%) and HCC or LREs in 5 (0.5%) cases (3-, 5-, 10-year cumulative rate: 0.2%, 0.5%, 0.5%). In this subgroup, the probability of HBsAg loss was independently associated with greater BMI (HR per kg/m^2: 1.15, 95% CI 1.06-1.25; p = 0.001) and lower baseline HBV DNA (HR per log_{10} IU/ml: 0.71, 95% CI 0.58-0.88; p = 0.002), while the probability of HCC or LREs was associated only with older age (HR per year: 1.09, 95% CI 1.01-1.18; p = 0.032) (Table S4). The probabilities of HBsAg loss, HCC and LREs in the 1,061 patients without a treatment indication or initiation during year 1 in relation to their ALT and/or HBV DNA levels during the same period are shown in Figs. S2 and S3.

The predictors of the main outcomes were also assessed with treated patients censored at treatment onset. It should be noted that 29/43 cases with HBsAg loss, 7/32 HCCs and 9/37

A

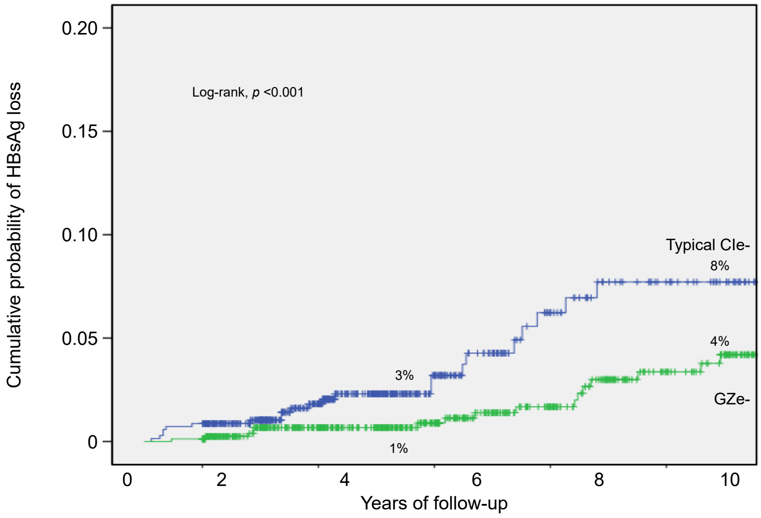

N° at risk						
Typical CIe-	690	594	320	170	118	95
GZe-	811	694	528	370	279	223

B

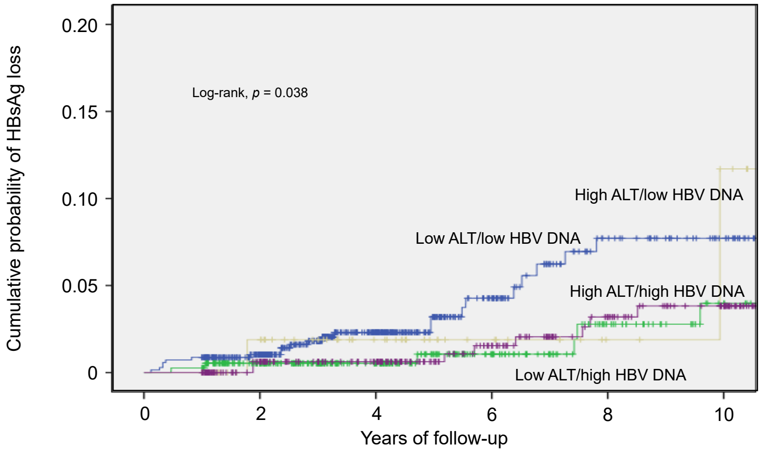

N° at risk						
Low ALT/low HBV DNA	690	594	320	170	118	95
High ALT/low HBV DNA	75	52	34	17	11	8
Low ALT/high HBV DNA	379	326	226	148	102	77
High ALT/high HBV DNA	357	316	268	203	166	138

Fig. 2. Probability of HBsAg loss in 1,501 HBeAg-negative patients classified according to baseline ALT and HBV DNA characteristics. Probability of HBsAg loss in 1,501 HBeAg-negative patients classified according to baseline ALT and HBV DNA characteristics into (A) chronic infection (CIe-) and grey-zone (GZe-) or (B) subgroups of ALT and HBV DNA levels. Kaplan-Meier curves were used for estimation of all cumulative rates, which were compared by log-rank test. ALT, alanine aminotransferase; CIe-, HBeAg-negative chronic infection; GZe-, HBeAg-negative grey-zone.

LREs developed in patients remaining untreated until the event. Using this approach, the probability of HBsAg loss was independently associated with higher BMI and lower baseline HBV DNA levels and the probability of both HCC and LREs was independently associated with older age and higher baseline HBV DNA levels (Table S5).

Discussion

GZe- or indeterminate patients with HBeAg-negative chronic HBV represent a heterogeneous group, as their definition varies widely across studies.[16] The definitions of GZe-patients may include different ALT and/or HBV DNA cut-off levels, as well as varying minimum durations and frequencies of follow-up, whereas they rarely include any assessment of liver histological severity.[6,7,16–24] Most previous studies included almost exclusively Asian patients, often both HBeAg positive and negative cases, who may have different natural history and thus different GZ criteria from Caucasian patients.[6,7,16,20–24] On the other hand, only HBVDNA cut-offs were used in one study from Italy[18] and combinations of ALT and HBVDNA cut-offs were used in one study from Spain.[19] In our study, HBeAg-negative patients were initially characterized according to their baseline ALT and HBV DNA levels, because we considered

Table 3. Multivariable Cox regression analyses for factors associated with the main outcomes in 1,501 HBeAg-negative patients.

	HBsAg loss<br>Adjusted HR (95% CI), *p* values	Hepatocellular carcinoma<br>Adjusted HR (95% CI), *p* values	Any LRE<br>Adjusted HR (95% CI), *p* values
Age, per year	NA	1.10 (1.06-1.15), <0.001	1.09 (1.05-1.13), <0.001
Sex, male *vs.* female	NA	3.55 (1.38-9.15), 0.009	1.99 (0.88-4.45) 0.096
Origin, Greece *vs.* abroad	NA	n.s.	n.s.
Body mass index, per kg/m^2	1.14 (1.06-1.22), 0.001	NA	NA
Diabetes, yes *vs.* no	NA	n.s.	n.s.
ALT, per IU/L	NA	NA	n.s.
AST, per IU/L	NA	n.s.	1.02 (1.00-1.03), 0.027
Platelets, per 10^3/mm^3	NA	0.98 (0.97-0.99), <0.001	0.990 (0.982-0.998), 0.019
HBV DNA, per log$_{10}$ IU/ml	NA	n.s.	n.s.
Liver stiffness, ≥8 *vs.* <8 kPa	NA	n.s.	n.s.
Baseline characteristics, grey-zone *vs.* chronic infection	0.51 (0.24-1.08), 0.078	36.35 (3.65-4912), <0.001	9.46 (1.13-79.54), 0.039
Treatment as time dependent variable	n.s.	n.s.	5.01 (0.87-28.91), 0.072

Main outcomes include HBsAg loss, hepatocellular carcinoma and any liver-related event.
ALT, alanine aminotransferase; AST, aspartate aminotransferase; HR, hazard ratio; NA, not applicable (*p* >0.10 in univariable Cox regression analysis); n.s., non-significant in multivariable Cox regression analysis (*p* >0.10).

such an approach to be closer to clinical practice. In addition, our patients were classified after the first year of follow-up when they were characterized as GZe- or CIe-cases based on EASL 2017[3] and our national guidelines. We also defined ALT/HBV DNA subgroups, similar to those used in other studies, to facilitate comparison of outcomes.[19] Using baseline data, 40% (811/2012) of all patients with HBeAg-negative chronic HBV seen at our tertiary liver center were found to have GZe- and 34% (690/2012) CIe-characteristics.

The incidence of treatment indications and the optimal management of GZe-patients remain controversial,[10] since variable proportions of them have been reported to have liver histological lesions of at least moderate severity justifying treatment initiation.[6,16,17,25,26] In our study, histological severity assessment by elastography or biopsy revealed treatment indications in 20% of patients with GZe- and 1.6% of patients with CIe-baseline characteristics, with the highest rates in those with ALT >1-2x ULN and HBV DNA >20,000 (37%). However, treatment indications by ALT/HBV DNA changes developed during year 1 in another 8.5% of our patients with GZe-baseline characteristics. Thus, better non-invasive markers might offer more accurate prediction of patients at risk of developing treatment indications in the near future. In any case, the fluctuating course of HBeAg-negative chronic HBV infection and especially the fluctuations of ALT and HBV DNA levels complicate follow-up, but also make it mandatory for these cases.[17] Of 1,061 patients without treatment indication/initiation during year 1, 11.5% of GZe- and 1.5% of CIe-patients developed treatment indications later. Moreover, during follow-up, 9.5% of CIe-patients developed GZ ALT/HBV DNA values, while 46% of untreated GZe-patients transitioned to a CIe-state. Several factors were identified to be independently associated with the development of treatment indications (Table 2), but no factor – including GZe- or CIe-status – could identify cases with little or no such risk. Thus, given that the 5-10-year cumulative probability of treatment indication was 35%-38% in GZe- and most importantly 5% in CIe-patients, long-term follow-up including periodic liver elastography is warranted for all HBeAg-negative cases.

The management of GZe-may differ among physicians and countries,[9,10,16] but such real-life data is not widely available. In fact, regardless of the development of treatment indications, physicians may decide to initiate treatment beyond the existing guidelines. This approach was clearly observed in our real-life study, as treatment initiation rates were higher than treatment indications rates in all subgroups. Almost 50% of patients with GZe-baseline characteristics started treatment within year 1 and the cumulative treatment rates approached 70%-80% at 5-10-year follow-up. As expected, treatment was initiated in almost all (97%) patients who developed treatment indications, but also in >40% of patients with GZe-characteristics at any time during follow-up. Given the complexity of long-term follow-up in daily clinical practice and the large proportion of GZe-patients who are eventually treated in real-life anyway, treatment may be justified in this setting.

Many studies have reported that, compared to CIe-, GZe-patients are at higher risk not only of developing treatment indications and progression to more severe fibrosis, but also of other complications including HCC.[6,7,16,24,26] Similar findings were observed in our cohort, as GZe-patients or those with GZe-baseline characteristics had significantly worse long-term outcomes, including HCC and LREs (cumulative rates: 3%-4% at year 5, 5%-6% at year 10), which were rarely observed in CIe-cases during the median 5-year follow-up. Except for GZe- or CIe-status, factors associated with the probability of adverse long-term outcomes were well known predictors like the PAGE-B parameters (age, sex, platelets) for HCC risk.[15] Moreover, patients with GZe-baseline characteristics had low cumulative probability of HBsAg loss (1% and 4% at year 5 and 10, respectively), which was higher but still numerically low in patients with CIe-baseline characteristics (3% and 8% at year 5 and 10, respectively). The probability of HBsAg loss, HCC, or LREs did not differ among the three GZe-baseline subgroups with high ALT and/or high HBV DNA; all three had a higher probability of HCC/LRE and, with the exception of the high-ALT/low-DNA subgroup, a lower probability of HBsAg loss compared with the CIe-group.

Although a proportion of GZe-patients may start treatment, usually with a NA in real life, the effect of treatment on their long-term outcomes has not been clarified. As already discussed, compared to CIe-, our GZe-patients had worse long-term outcomes despite more frequent treatment initiation. Furthermore, treatment initiation was not associated with the probability of HCC and showed a trend toward a negative independent association with LRE development when treatment

A

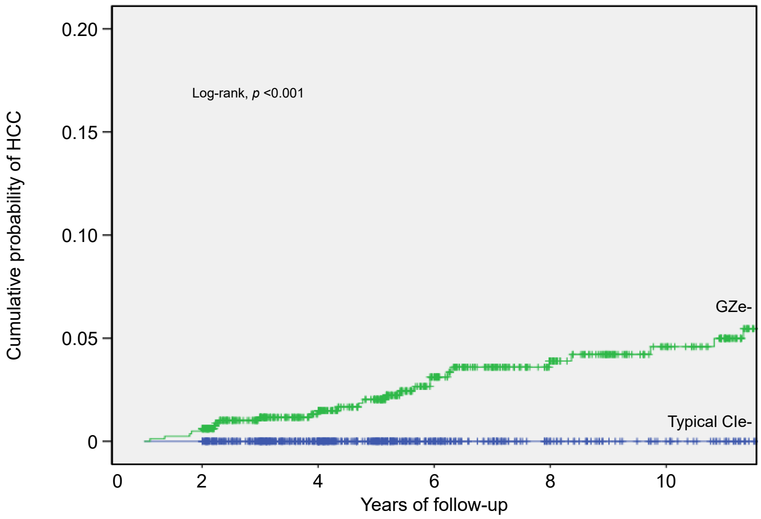

N° at risk						
Typical CIe-	690	599	328	175	121	98
GZe-	811	688	524	367	281	227

B

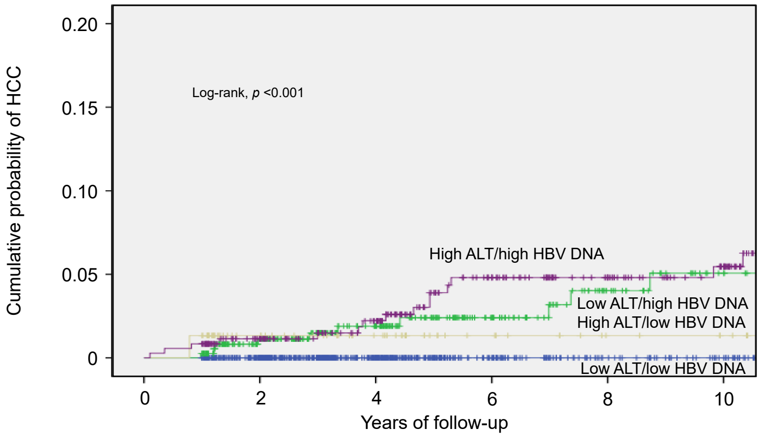

N° at risk						
Low ALT/low HBV DNA	690	599	328	175	121	97
High ALT/low HBV DNA	75	52	35	19	11	8
Low ALT/high HBV DNA	379	323	224	148	101	79
High ALT/high HBV DNA	357	313	265	200	168	140

Fig. 3. Probability of HCC in 1,501 HBeAg-negative patients classified according to baseline ALT and HBV DNA characteristics. Probability of HCC in 1,501 HBeAg-negative patients classified according to baseline ALT and HBV DNA characteristics into (A) chronic infection (CIe-) and grey-zone (GZe-) or (B) subgroups of ALT and HBV DNA levels. Kaplan-Meier curves were used for estimation of all cumulative rates, which were compared by log-rank test. ALT, alanine aminotransferase; CIe-, HBeAg-negative chronic infection; GZe-, HBeAg-negative grey-zone; HCC, hepatocellular carcinoma.

was analyzed as a time-dependent variable. It should be noted, however, that no valid conclusions can be drawn about the effect of treatment on long-term outcomes in this setting based on real-world data, since patients with GZe-*vs.* CIe-baseline characteristics, as well as treated *vs.* untreated GZe-patients, differ significantly in several strong predictors of LREs that cannot be fully accounted for by statistical analysis. Moreover, since a proportion of our GZe-patients remained under follow-up without treatment for variable periods, a potential treatment effect in case of earlier treatment onset cannot be excluded.

Our study has certain limitations. First, as treatment initiation was decided by six attending physicians, no uniform criteria were applied and treatment was initiated regardless of treatment indications. That said, treatment initiation rates probably better reflect the current management of HBeAg-negative chronic HBV, at least in daily clinical practice in Greece. At the same time, we attempted to minimize the limitations in assessing the treatment effect by using Cox regression analyses with time-dependent models, as well as by censoring treated patients at the time of treatment initiation. Second, as discussed, the potential effect of treatment

A

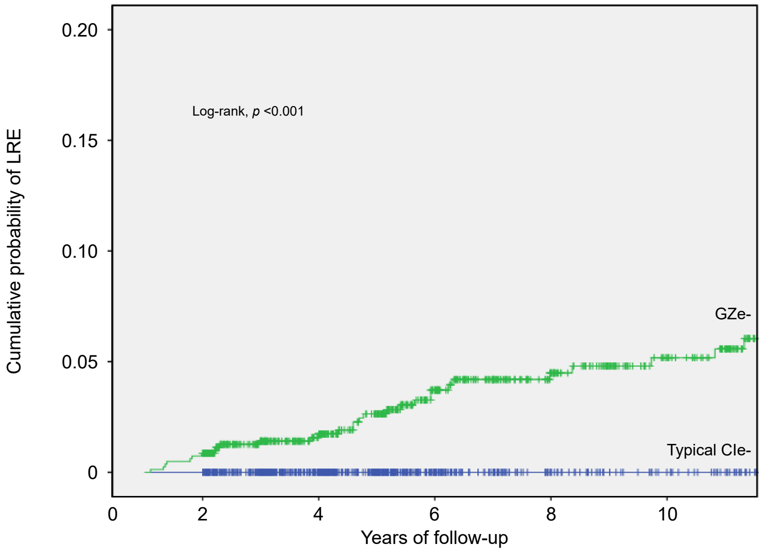

N° at risk						
Typical CIe-	690	599	328	174	121	98
GZe-	811	686	524	367	281	227

B

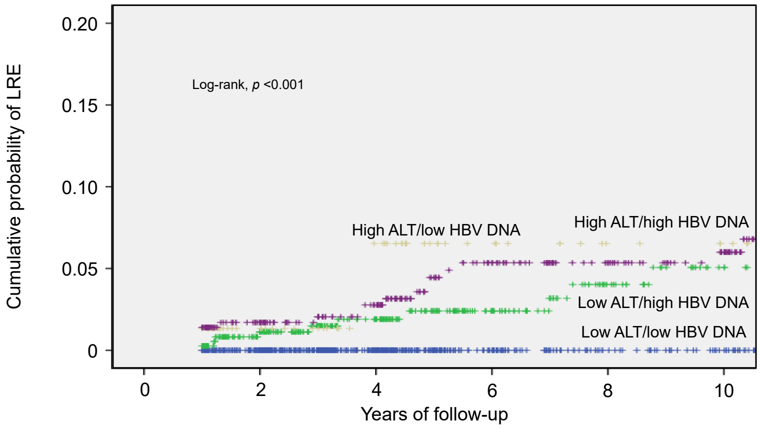

N° at risk						
Low ALT/low HBV DNA	690	599	328	175	121	97
High ALT/low HBV DNA	75	52	35	19	11	8
Low ALT/high HBV DNA	379	323	224	148	102	78
High ALT/high HBV DNA	357	311	265	200	168	140

Fig. 4. Probability of LREs in 1,501 HBeAg-negative patients classified according to baseline ALT and HBV DNA characteristics. Probability of LREs in 1,501 HBeAg-negative patients classified according to baseline ALT and HBV DNA characteristics into (A) chronic infection (CIe-) and grey-zone (GZe-) or (B) subgroups of ALT and HBV DNA levels. LREs included hepatocellular carcinoma, decompensated cirrhosis, liver transplantation and liver-related death. Kaplan-Meier curves were used for estimation of all cumulative rates, which were compared by log-rank test. ALT, alanine aminotransferase; CIe-, HBeAg-negative chronic infection; GZe-, HBeAg-negative grey-zone; LREs, liver-related events.

on the development of major outcomes cannot be reliably assessed in our non-randomized cohort of treated and untreated GZe-patients, who differ significantly in all major predictors of outcomes. Third, our study may not have the power to detect a potential difference in outcomes of low probability, such as HCC and LRE, in a cohort followed for a limited duration for such events. Fourth, our findings are limited to Caucasian patients; therefore, the conclusions cannot be extrapolated to countries with different patient populations or HBV genotypes, nor to other centers managing similar patients using different approaches. Fifth, unfortunately information on specific markers which could improve the classification and prediction of patients, such as quantitative HBsAg and hepatitis B core related antigen, was not available in our study.

In conclusion, GZe-patients or those with GZe-baseline characteristics represent a large proportion of all patients with chronic HBV. Compared to patients with CIe-, GZe-patients have lower probability of HBsAg loss and higher risk for worse long-term outcomes, including HCC and LREs, despite more frequent treatment initiation. Given the higher risk of adverse long-term outcomes, which aligns with the

frequent presence of negative predictors at baseline, and since physicians often initiate treatment in the majority of GZe-patients – even in the absence of treatment indications – the optimal management and timing of treatment initiation require further evaluation in this setting; however, starting treatment in GZe-patients may be justified. The newly released EASL HBV guidelines further support such an approach.[27]

Affiliations

[1] *1st Department of Gastroenterology, Medical School of National & Kapodistrian University of Athens, General Hospital of Athens "Laiko", Athens, Greece;* [2] *1st Department of Pathology, Medical School of National & Kapodistrian University of Athens, General Hospital of Athens "Laiko", Athens, Greece*

Abbreviations

ALT, alanine aminotransferase; AST, aspartate aminotransferase; CIe-, HBeAg-negative chronic infection; EASL, The European Association for the Study of the Liver; GZ, grey-zone; GZe-, HBeAg-negative grey-zone; HCC, hepatocellular carcinoma; LREs, liver-related events; NA, nucleos(t)ide analogue; ULN, upper limit of normal.

Financial support

The study was supported by an unrestricted grant from Gilead Sciences and by Hellenic Foundation of Gastroenterology and Nutrition.

Conflicts of interest

M Papatheodoridi, S Paraskevopoulou, P Ioannidou, P Fytili, D Karagiannakis, A Papatheodoridi and E Cholongitas have nothing to disclose. S Sakellariou has served as lecturer for Roche. J Vlachogiannakos has served as advisor and/or lecturer for Abbvie, Astra-Zeneca, Bayer, BioArs, Galenica, Gilead, Integris Pharma, Sobi and Viatris. G Papatheodoridis has served as advisor and/or lecturer for Abbvie, Albireo, Amgen, Astra-Zeneca, BioArs, Elpen, Genesis, Gilead, GlaxoSmithKline, Ipsen, Janssen, Merck Sharp & Dohme, Novo Nordisk, Roche, Takeda and Vir Pharmaceuticals and has received research grants from Abbvie, Gilead, Takeda and Vianex.

Please refer to the accompanying ICMJE disclosure forms for further details.

Authors' contributions

M Papatheodoridi contributed to the study conception, study design and data collection, performed statistical analyses and prepared the first draft of the manuscript. S Paraskevopoulou, P Ioannidou, P Fytili, DS Karagiannakis, E Cholongitas and I Vlachogiannakos contributed to the study design and data collection and made critical revisions of the manuscript. A Papatheodoridi contributed to data collection and statistical analyses and made critical revisions of the manuscript. S Sakellariou was responsible for histological assessments and made critical revisions of the manuscript. G Papatheodoridis contributed to the study conception, study design and statistical analyses and made critical revisions of the manuscript. All authors read and approved the final manuscript.

Data availability

All data can be available from the corresponding author upon reasonable request.

Acknowledgements

We thank Mr Sotirios Roussos for his support in statistical analyses.

Supplementary data

Supplementary data to this article can be found online at https://doi.org/10.1016/j.jhepr.2026.101771.

References

Author names in bold designate shared co-first authorship

[1] Hadziyannis SJ, Papatheodoridis GV. Hepatitis Be antigen negative chronic hepatitis B – natural history and treatment. Semin Liver Dis 2006;26:130–141.
[2] Papatheodoridis GV, Manolakopoulos S, Dusheiko G, et al. Therapeutic strategies in the management of patients with chronic hepatitis B. Lancet Infect Dis 2008;8:167–178.
[3] European Association for the Study of the Liver. EASL 2017 Clinical Practice Guidelines on the management of hepatitis B virus infection. J Hepatol 2017;67:370–398.
[4] Terrault NA, Lok ASF, McMahon BJ, et al. Update on prevention, diagnosis, and treatment of chronic hepatitis B: AASLD 2018 hepatitis B guidance. Hepatology 2018;67:1560–1599.
[5] Sarin SK, Kumar M, Lau GK, et al. Asian-Pacific clinical practice guidelines on the management of hepatitis B: a 2015 update. Hepatol Int 2016;10:1–98.
[6] **Yao K**, **Liu J**, **Wang J**, et al. Distribution and clinical characteristics of patients with chronic hepatitis B virus infection in the grey zone. J Viral Hepat 2021;28:1025–1033.
[7] **Huang DQ**, **Li X**, Le MH, et al. Natural history and hepatocellular carcinoma risk in untreated chronic hepatitis B patients with indeterminate phase. Clin Gastroenterol Hepatol 2022;20:1803–1812.
[8] **Gan QY**, **Wang JX**, **Qian F**, et al. Clinical and histological features of patients with chronic hepatitis B virus infection in the grey zone. J Viral Hepat 2023;30:803–809.
[9] **Tan M**, **Bhadoria AS**, Cui F, et al. Estimating the proportion of people with chronic hepatitis B virus infection eligible for hepatitis B antiviral treatment worldwide: a systematic review and meta-analysis. Lancet Gastroenterol Hepatol 2021;6:106–119.
[10] **Sheng Q**, **Wang N**, Zhang C, et al. HBeAg-Negative patients with chronic hepatitis B virus infection and normal alanine aminotransferase: wait or treat? J Clin Transl Hepatol 2022;10:972–978.
[11] World Health Organization. Guidelines for the prevention, diagnosis, care and treatment for people with chronic hepatitis B infection. https://www.who.int/publications/i/item/9789240090903.2024.March.29.
[12] You H, Wang F, Li T, et al. Guidelines for the prevention and treatment of chronic hepatitis B (version 2022). J Clin Transl Hepatol 2023;11:1425–1442.
[13] European Association for the Study of the Liver. Asociacion Latinoamericana para el Estudio del Higado. EASL-ALEH Clinical Practice Guidelines: Non-invasive tests for evaluation of liver disease severity and prognosis. J Hepatol 2015;63:237–264.
[14] European Association for the Study of the Liver. EASL clinical practice guidelines: management of hepatocellular carcinoma. J Hepatol 2018;69:182–236.
[15] Papatheodoridis G, Dalekos G, Sypsa V, et al. PAGE-B predicts the risk of developing hepatocellular carcinoma in Caucasians with chronic hepatitis B on 5-year antiviral therapy. J Hepatol 2016;64:800–806.
[16] **Lai JC**, **Wong GL**, Tse YK, et al. Histological severity, clinical outcomes and impact of antiviral treatment in indeterminate phase of chronic hepatitis B: a systematic review and meta-analysis. J Hepatol 2025;82:992–1003.
[17] Papatheodoridis GV, Manolakopoulos S, Liaw Y-F, et al. Follow-up and indications for liver biopsy in HBeAg-negative chronic hepatitis B virus infection with persistently normal ALT: a systematic review. J Hepatol 2012;57:196–202.
[18] Oliveri F, Surace L, Cavallone D, et al. Long-term outcome of inactive and active, low viraemic HBeAg-negative-hepatitis B virus infection: benign course towards HBsAg clearance. Liver Int 2017;37:1622–1631.
[19] Bonacci M, Lens S, Marino Z, et al. Anti-viral therapy can be delayed or avoided in a significant proportion of HBeAg-negative Caucasian patients in the Grey Zone. Aliment Pharmacol Ther 2018;47:1397–1408.
[20] Zhou K, Wahed AS, Cooper S, et al. Phase transition is infrequent among North American adults with e-antigen-negative chronic hepatitis B and low-level viremia. Am J Gastroenterol 2019;114:1753–1763.
[21] Lok AS, Perrillo R, Lalama CM, et al. Low incidence of adverse outcomes in adults with chronic hepatitis B virus infection in the era of antiviral therapy. Hepatology 2021;73:2124–2140.
[22] Huang R, Do AT, Toyoda H, et al. Distribution, characteristics, and natural history of diverse types of indeterminate chronic hepatitis B: a REAL-B study. Aliment Pharmacol Ther 2025;62:349–358.
[23] Huang R, Trinh HN, Yasuda S, et al. Differential HCC risk among HBV indeterminate types at baseline and by phase transition. Gut 2025;74:1873–1882.
[24] Hui VW, Wong GL, Dai J, et al. Increased risk of HCC in HBeAg-negative indeterminate phase compared with HBeAg-negative chronic infection. Hepatology 2026;83:156–168.

[25] Papatheodoridis GV, Manesis EK, Manolakopoulos S, et al. Is there a meaningful serum HBV DNA cut-off level for therapeutic decisions in HBeAg-negative chronic hepatitis B virus infection? Hepatology 2008;48:1451–1459.
[26] Huang DL, Cai QX, Zhou GD, et al. Liver histological changes in untreated chronic hepatitis B patients in indeterminate phase. World J Hepatol 2024;16:920–931.
[27] European Association for the Study of the Liver. EASL Clinical Practice Guidelines on the management of hepatitis B virus infection. J Hepatol 2025;May 8. https://doi.org/10.1016/j.jhep.2025.03.018. S0168-8278(25)00174-6.

Keywords: inactive carrier; liver elastography; liver biopsy.
Received 19 October 2025; received in revised form 21 January 2026; accepted 29 January 2026; Available online 4 February 2026

Journal of Hepatology, Volume *8*

Supplemental information

Long-term outcomes of baseline grey-zone patients with HBeAg-negative chronic hepatitis B virus infection

Margarita Papatheodoridi, Sofia Paraskevopoulou, Panagiota Ioannidou, Paraskevi Fytili, Dimitrios S. Karagiannakis, Alkistis Papatheodoridi, Stratigoula Sakellariou, Evangelos Cholongitas, Ioannis Vlachogiannakos, and George Papatheodoridis

Long-term outcomes of baseline grey-zone patients with HBeAg-negative chronic hepatitis B virus infection

Margarita Papatheodoridi, Sofia Paraskevopoulou, Panagiota Ioannidou, Paraskevi Fytili, Dimitrios S Karagiannakis, Alkistis Papatheodoridi, Stratigoula Sakellariou, Evangelos Cholongitas, Ioannis Vlachogiannakos, George Papatheodoridis

Table of contents

Fig. S1…………………………………………………………………………..…2

Fig. S2……………………………………………………………………………..4

Fig. S3……………………………………………………………………………..5

Table S1…………………………………………………………………………...7

Table S2…………………………………………………………………………...8

Table S3………………………………………………………………………... 10

Table S4…………………………………………………………………..……...11

Table S5………………………………………………………………….........13

Fig. S1. Probability of development of (A) treatment indication or (B) treatment initiation in 1501 patients with HBeAg-negative chronic HBV infection classified in relation to their baseline ALT and/or HBV DNA levels into chronic infection (CIe-: ALT≤ULN and HBVDNA<2000 IU/mL) and grey-zone (GZe-: HBVDNA <2,000 IU/mL and ALT>ULN, HBVDNA 2,000-20,000 IU/mL, or HBVDNA>20,000 IU/mL and ALT<2xULN). All treatments starting during the first year of follow-up were considered to start at year 1. Kaplan-Meier curves were used for estimation of all cumulative rates, which were compared by log-rank test.

Fig. 1A.

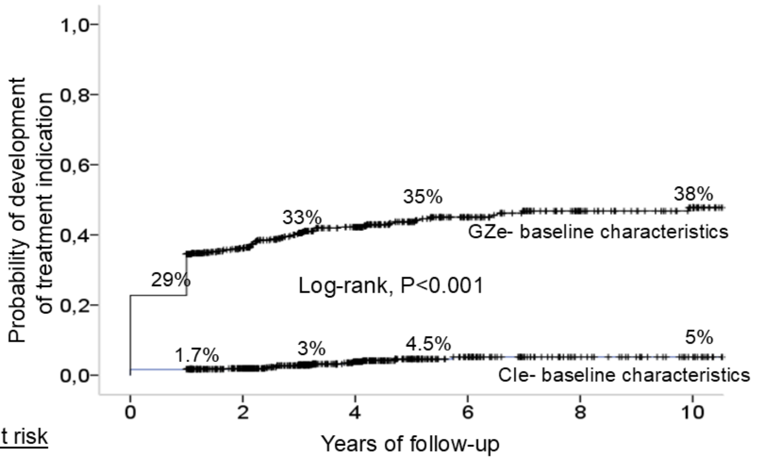

Patients at risk						
CIe-	690	590	315	163	112	90
GZe-	811	469	320	194	131	100

Suppl. Fig. 1A

Fig 1B.

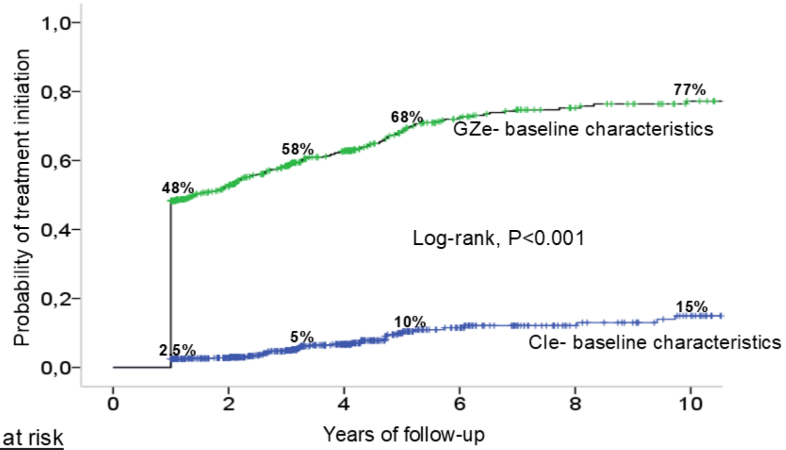

Patients at risk

Cle-	690	586	304	151	102	82
GZe-	811	322	173	77	44	27

Suppl. Fig. 1B

Fig. S2. Probability of HBsAg loss in 1061 patients with HBeAg-negative chronic HBV infection without treatment indication or initiation during the 1st year in relation to their ALT and/or HBV DNA levels during the same period. Kaplan-Meier curves were used for estimation of all cumulative rates, which were compared by log-rank test.

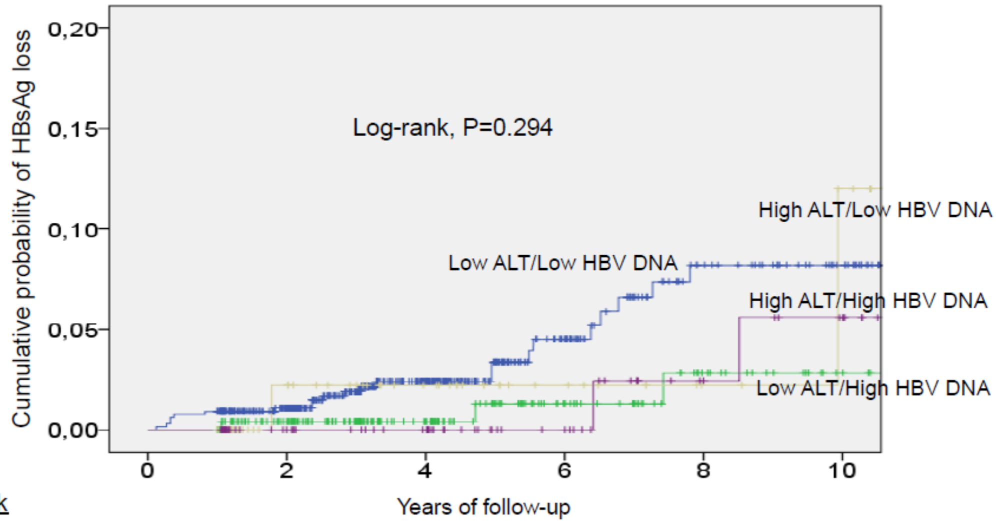

Patients at risk

Low ALT/Low HBV DNA	658	569	301	158	109	88
High ALT/Low HBV DNA	67	44	31	18	11	8
Low ALT/High HBV DNA	246	208	106	85	57	43
High ALT/High HBV DNA	90	74	63	46	32	27

Fig. S3. Probability of (A) hepatocellular carcinoma (HCC) or (B) liver-related event (LRE) development in 1061 patients with HBeAg-negative chronic HBV infection without treatment indication or initiation during the 1st year in relation to their ALT and/or HBV DNA levels during the same period. LRE included HCC, decompensated cirrhosis, liver transplantation and liver-related death. Patients with Low ALT/Low HBV DNA and High ALT/Low HBV DNA as well as those with Low ALT/High HBV DNA and High ALT/High HBV DNA patients had the same numbers and the same cumulative probabilities of HCC and LRE. Kaplan-Meier curves were used for estimation of all cumulative rates, which were compared by log-rank test.

Fig 3A

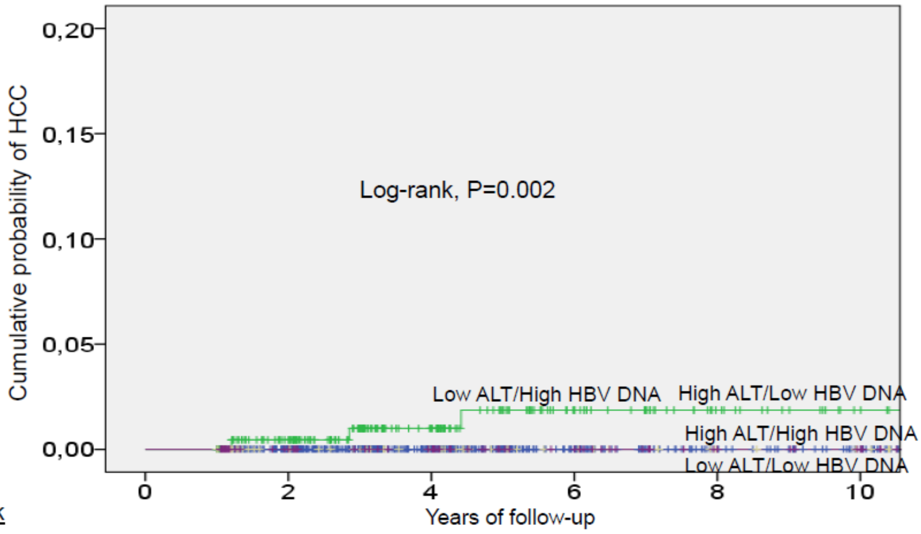

Patients at risk	0	2	4	6	8	10
Low ALT/Low HBV DNA	658	574	309	163	112	91
High ALT/Low HBV DNA	67	45	32	18	11	8
Low ALT/High HBV DNA	246	214	137	86	59	45
High ALT/High HBV DNA	90	75	63	46	33	29

Fig 3B

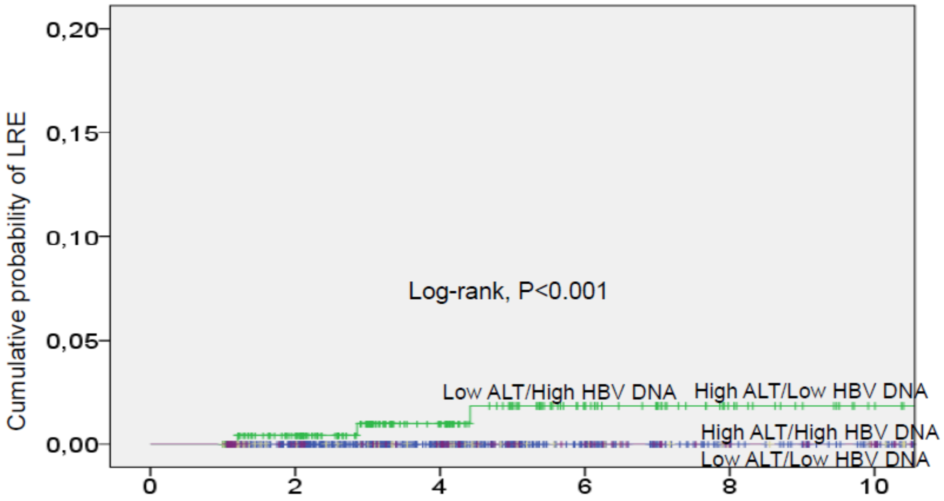

Patients at risk

Low ALT/Low HBV DNA	658	574	308	163	112	91
High ALT/Low HBV DNA	67	45	32	18	11	8
Low ALT/High HBV DNA	246	214	137	86	59	45
High ALT/High HBV DNA	90	73	63	46	33	29

Table S1. Liver fibrosis severity by liver elastography (n=1499) and/or liver biopsy (n=283*) in 1501 HBeAg-negative chronic hepatitis B patients.

Liver stiffness, kPa	**All patients** n (%)	**Patients with liver biopsy*** - Ishak's stage, n (%)				
		0	1	2	3-4	5-6
≤6.4	910 (60.7)	9 (75.0)	45 (68.2)	38 (48.7)	10 (10.3)	0
6.5-7.9	303 (20.2)	3 (25.0)	16 (24.2)	22 (28.2)	27 (27.6)	0
8-9	102 (6.8)	0	4 (6.1)	8 (10.3)	15 (15.5)	0
9.1-12 for ALT>ULN	63 (4.2)	0	1 (1.5)	7 (9.0)	20 (20.6)	2 (7.1)
9.1/12-12.1/15 for ALT≤/>ULN	50 (3.3)	0	0	2 (2.6)	15 (15.5)	0
>12/15 for ALT≤/>ULN	71 (4.7)	0	0	1 (1.3)	10 (10.3)	26 (92.9)
Total	1499 (100)	12 (4.2)	66 (23.5)	78 (27.8)	97 (34.5)	28 (10.0)

*Two patients had a liver biopsy (Ishak's stage: 2 and 6) but not elastography.

ULN: upper limit of normal.

Table S2. Subgroups of 1501 HBeAg-negative chronic hepatitis B (CHBVe-) patients according to their serum HBVDNA and ALT levels at baseline and maximum values during the first year of follow-up.

Maximum HBVDNA (IU/mL) and ALT levels during year-1		Groups of CHBVe- patients by baseline ALT and HBVDNA (IU/mL) levels, n (%)							
		HBVDNA<2,000			HBVDNA-=2,000-20,000			HBVDNA>20,000	
		ALT<ULN	ALT>1-2xULN	ALT>2xULN	ALT<ULN	ALT>1-2xULN	ALT>2xULN	ALT<ULN	ALT>1-2xULN
HBVDNA <2,000	ALT<ULN	677 (45.1)	0	0	0	0	0	0	0
	ALT>1-2xULN	1 (0.1)	58 (3.9)	0	0	0	0	0	0
	ALT>2xULN	0	2 (0.1)	10 (0.7)	0	0	0	0	0
HBVDNA= 2,000-20,000	ALT<ULN	7 (0.5)	0	0	259 (17.3)	0	0	1 (0.1)	0
	ALT>1-2xULN	4 (0.3)	4 (0.3)	0	7 (0.5)	76 (5.1)	0	0	0
	ALT>2xULN	1 (0.1)	0	1 (0.1)	2 (0.1)	10 (0.7)	47 (3.1)	1 (0.1)	1

HBVDNA >20,000	ALT<ULN	0	0	0	1 (0.1)	0	0	54 (3.6)	0
	ALT>1-2xULN	0	0	0	14 (0.9)	10 (0.7)	0	18 (1.2)	131 (8.7)
	ALT>2xULN	0	0	0	8 (0.5)	9 (0.6)	18 (1.2)	14 (0.9)	56 (3.7)
Total		690 (46.0)	64 (4.3)	11 (0.7)	291 (19.4)	105 (7.0)	65 (4.3)	88 (5.9)	187 (12.5)

ULN: upper limit of normal

Table S3. Development of treatment indications and treatment initiation during the first year of follow-up in 1501 HBeAg-negative chronic hepatitis B patients according to their maximum serum HBVDNA and ALT levels during the same period. Treatment indication was considered to be met in patients with a) ALT>2xULN and HBVDNA>20,000 IU/mL regardless of histological severity, b) HBVDNA>2,000 IU/mL and advanced fibrosis regardless of ALT and c) detectable HBVDNA and cirrhosis.

Maximum HBVDNA (IU/mL) and ALT levels during year-1		Treatment indication, n/N (%)	Treatment initiation, n (%)
HBVDNA <2,000	ALT≤ULN	10/677 (1.5)	14/677 (2.1)
	ALT>1-2xULN	1/59 (1.7)	2/59 (3.4)
	ALT>2xULN	1/12 (8.3)	2/12 (16.7)
HBVDNA= 2,000-20,000	ALT≤ULN	21/267 (7.9)	37/267 (13.9)
	ALT>1-2xULN	20/91 (22.0)	28/91 (30.8)
	ALT>2xULN	26/62 (41.9)	49/62 (79.0)
HBVDNA >20,000	ALT≤ULN	11/55 (20.0)	25/55 (45.5)
	ALT>1-2xULN	50/173 (28.9)	147/173 (75.0)
	ALT>2xULN	105/105 (100.0)	105/105 (100.0)
Total		245/1501 (16.3)	409/1501 (27.2)

ULN: upper limit of normal

Table S4. Multivariable Cox regression analyses for factors associated with the main outcomes in in 1061 patients with HBeAg-negative chronic HBV infection (CHBVe-) without treatment indications and initiation during year-1. Main outcomes include HBsAg loss, hepatocellular carcinoma and any liver related event (LRE).

	**HBsAg loss**	**Hepatocellular carcinoma or LRE**
	Adjusted HR (95% CI), P	Adjusted HR (95% CI), P
Age, per year	NA	1.09 (1.01-1.18), 0.032
Gender, male vs female	NA	NA
Origin, Greece vs abroad	NA	NS
Body mass index, per kg/m^2	1.15 (1.06-1.25), 0.001	NA
Diabetes, yes vs no	NA	NA
ALT, per IU/L	NA	NA
AST, per IU/L	NA	NA
Platelets, per 10^3/mm^3	NA	NA
HBVDNA, per $\log_{10}$ IU/mL	0.71 (0.58-0.88), 0.002	NS

CHBVe- group, Grey-Zone vs Chronic infection	NA	NA
Treatment as time dependent variable	NA	NS

HR: Hazard ratio, CI: confidence interval, ALT: alanine aminotransferase, AST: aspartate aminotransferase, NA: not applicable (P>0.10 in univariable Cox regression analysis); NS: non-significant in multivariable Cox regression analysis (P>0.10).

Table S5. Multivariable Cox regression analyses for baseline factors associated with the main outcomes in 1501 patients with HBeAg-negative chronic HBV infection (CHBVe-). Main outcomes include HBsAg loss, hepatocellular carcinoma and any liver related event (LRE). Patients were censored at the onset of treatment.

	**HBsAg loss**	**Hepatocellular carcinoma**	**Any LRE**
	Adjusted HR (95% CI), P	Adjusted HR (95% CI), P	Adjusted HR (95% CI), P
Age, per year	NA	1.08 (1.02-1.15), 0.015	1.09 (1.02-1.17), 0.011
Gender, male vs female	NA	NA	NA
Origin, Greece vs abroad	NA	NA	NA
Body mass index, per kg/m^2	1.17 (1.07-1.27), 0.001	NA	NA
Diabetes, yes vs no	NA	NA	NS
ALT, per IU/L	NA	NA	NS
AST, per IU/L	NA	NA	NS
Platelets, per $10^3/mm^3$	NA	NS	NS
HBVDNA, per log_{10} IU/mL	0.64 (0.48-0.75), 0.002	2.06 (1.20-3.52), 0.009	1.65 (1.03-2.64), 0.036

CHBVe- group, Grey-Zone vs Chronic infection	NS	NA	NA

HR: Hazard ratio, CI: confidence interval, ALT: alanine aminotransferase, AST: aspartate aminotransferase, NA: not applicable (P>0.10 in univariable Cox regression analysis); NS: non-significant in multivariable Cox regression analysis (P>0.10).
